# Digital storytelling as student-centred pedagogy: empowering high school students to frame their futures

**DOI:** 10.1186/s41039-017-0061-9

**Published:** 2017-11-21

**Authors:** Bea Staley, Leonard A. Freeman

**Affiliations:** Charles Darwin University, Darwin, Australia

**Keywords:** Digital storytelling, Student-centred learning, Rural, Youth, High school, Future, Aspiration

## Abstract

Digital storytelling was used in a high school classroom in the Midwestern USA as a part of the curriculum for “non-university-bound” rural youth. Though described as “unengaged”, in this paper we illustrate the way this digital storytelling project redefined the teacher-student power relationship, and students responded by producing work that was opinionated, forceful and demonstrated a thorough engagement with academic practices via technologies.

Research demonstrates that teacher expectations impact student outcomes, and for marginalised students, it is essential to provide pedagogical opportunities that affirm the student’s culture and identity. In this paper, we describe the project and the ways students talked about their education and their future through their digital stories. We use Smyth’s (International Journal of Leadership in Education 9(4):285–298, 2006) learner-centred policy constellation to consider the findings, and reframe the way we view these students and their work.

By utilising technologies in a meaningful way in the classroom, we anticipate educators can potentially deliver more effective, powerful and engaging pedagogies to all students, including those on nonmainstream educational pathways.

## Introduction

Digital storytelling is a form of personal storytelling, which simultaneously combines voice, image and printed text to tell a short—typically 3- to 5-min—focused story (e.g. Lambert, 2009; Ohler, 2010; Robin, 2008). This paper describes one digital storytelling project, selected as a method for its “democratic potential” (Couldry, 2008) and enacted with a group of white, rural American high school students described by teaching staff as “unmotivated, low functioning sometimes for reasons they can’t control or we don’t even know about” with “major behaviour issues” (field notes, September 4, 2012). Using a workshop method, digital storytelling provides a way for people to harness modern technologies to share experiences of their daily lives. In this work we are referring both to digital story telling as a specific form and practice^1^ (Hartley & McWilliam, 2009) that has grown out of Lambert’s (2010) work at the Center for Digital Storytelling^2^ in the USA.

Rural students are often marginalised in the dialogue about public school students in the USA. Yet, we illustrate here that working with student stories via digital storytelling offered an opportunity for this classroom teacher to engage in pedagogical activities which included critiquing and challenging normative and restrictive expectations, beliefs and values imposed upon rural students. We share this student work to illustrate how we can use digital between stories to consider how students access opportunities, confront roadblocks and name educational inequities within their school, in ways that encourage them to both determine and question their own goals and future aspirations.

In this research, students completed digital stories and engaged with the theme of their “future”. There are two words of note in this sentence; one is *future*, as these students were often seen as future*less* within the school system because they were not headed to university. The second word to highlight is *engaged* as the digital storytelling project was successfully completed by this group, and they demonstrated a capacity often overlooked in their other school-based assignments. In this paper we share the story of the project, the stories of the students, and discuss how technology can be used to harness student’s interests and skills. Smyth’s (2006) learner-centred policy constellation is used to frame our discussion regarding the suitability of using digital storytelling as a technology with marginalised high school students to shift the classroom dynamics from teacher-centred instruction to student-centred learning.

This work has three premises: One, that we should seek to provide students with an education that allows them to consider and pursue a meaningful life based on their own inherent interests, talents and aspirations (Sizer, 1996; Quaglia & Cobb, 1996). Two, that storytelling has the potential to be transformative (e.g. Bell, 2010). Three, that educators have a responsibility to not only teach, but to contextualise the learning for students so they appreciate the content and its potential relevance to their lives (Lee, 2007). We demonstrate that technologies, digital storytelling in particular, can be utilised as a tool to support these guiding assumptions.

In this work we apply critical literacy theory to the teaching context of white, rural youth, to evaluate the effectiveness of utilising digital storytelling as a tool to reposition the power relations between teacher and students. A learner-centred framework is utilised in our discussion to privilege the students’ voices, as they grapple with issues of identity and become the narrators of their own stories about their lives and future aspirations.

## Methods

### The class and students

This research took place at Shady Grove High School, a public school with 629 students (> 98% Caucasian) in grades 9 through 12, with 33 fulltime educators. The school has a rural-fringe designation as assigned by the US Census bureau^3^ and the Midwestern state where this research takes place has the fourth largest rural school enrolment in the USA (Strange et al. 2012).

The 49 students that participated in this digital storytelling project were enrolled in a class called *Journey Beyond Shady Grove* instituted by the school board for their “non-university-bound” students in lieu of taking *Journey to University*. The *Journey to University* curriculum included crucial aspects of applying and going to university. *Journey Beyond Shady Grove* had a more fluid directive about life beyond high school and was designed by the classroom teacher Ms. Lane,^4^ to “give the students the skills they need to get on with their life” (field notes, August 27, 2012). This research and the digital storytelling project was incorporated into the curriculum with approval of the principal who connected the teacher and principal researcher prior to the onset of the semester.^5^

At Shady Grove High School, the curricular emphasis was firmly on higher education. The school superintendent gave a talk to the class, where he asked, “do you think you are prepared academically for university? I hope so. That’s the goal, that’s what we are trying to accomplish” (field notes, November 15, 2012). It is notable that this is a stance that disenfranchises almost 50% of their graduating student body, as 48% of the students do not go on to 4-year colleges (principal meeting, field notes).

That said, the course description (for “non-university-bound” students) was likely a misnomer, as the class was a catch-all for any students not taking *Journey to University*. For example, Ted’s story noted his intention to join the Marine Corps after high school, and 15% of the students (7 of 30 males enrolled in *Journey Beyond Shady Grove*) were bound for the military.

When asked to describe themselves, the students in this work told us “we’re not real preppy. We’re all good old boys.” They believed people saw them as “a bunch of hicks… like rednecks, like farms and tractors” and thought this was a mostly true description (field notes, September 25, 2012). Within this student group, they self-reported histories of abuse, experiencing the death of a loved one (e.g. mothers, cousins, boyfriends), and parental abandonment at a young age. Students told of tenuous relationships with the school administration. They reported a love of hunting, football, fishing and being outside. Some of the students in this group required educational support services. Some simply did not come to school very often; many of these students presented with (and reported) a withdrawn or disengaged affect at school.

### The digital story approach

Digital stories have been increasingly used in classrooms (Ohler, 2013) and their educational benefits include student engagement, access to broader audiences, amplification of individual voices and the harnessing of student emotion (Lowenthal, 2009; Opperman, 2008; Selfe & Selfe, 2008). Digital storytelling can also be used as a method for engaging in dialogue with students about important community issues as they develop, refine and produce personal stories that have the potential to impact self and others (see Hull, 2003; Lambert, 2009; Taub-Pervizpour, 2009). Further, digital storytelling provides an opportunity for engaging in multiple literacies, and the utilisation of varied technologies. The tasks inherent in the digital storytelling process offered youth the chance to learn new skills and demonstrate their proficiency in a multitude of programs (such as iMovie, Creative Commons Search and Jamendo).

Digital storytelling was also used, because telling stories is useful for centralising students in their learning and giving them the power to explain and position themselves in their lives and education. Nieto (1994) writes, “one way to begin the process of changing school policies and practices is to listen to students’ views about them” (p. 396). Here, digital storytelling was used to explore adolescents’ reflections on schooling. The stories gathered and shared in this article contribute to the “knowing” of heterogeneous youth viewpoints (D’Amico et al., 1996; Panelli, 2002). That youth are creators and innovators is an underlying assumption of this work (Kinloch, 2012; Morrell, 2008), and as such their digital stories and contributions provide valuable insight into contemporary issues in education.

The digital storytelling approach used in this research was based on Lambert’s (2010) Digital Storytelling Cookbook, because the researcher had attended a digital storytelling workshop based on the workshop method developed by Lambert and the Center for Digital Storytelling^6^. This method was adapted for the classroom setting and the time constraints of the school schedule. When this research was proposed to the school principal, the administration had recently implemented a netbook program which meant every student had a new school laptop (netbook) and these individual computers were all linked to a central system. At the time the administration and faculty were interested in ways that they could increasingly engage technologies across the curriculum. It should be noted that these students had varying proficiency with this new equipment and in subsequent interviews both students and educators reported this would all be easier in the future when students receive their netbook in the first year of high school. Most of these students did not have the technological literacy or skills we might expect from contemporary students, who are often referred to as “digital natives” (Prensky, 2001).

### Creating digital stories

The digital storytelling project was presented to the students at the beginning of semester. From the outset, students were fairly receptive to the project overall and the relative completion rates would suggest this was a successful project for this student group. The students created their digital stories over a period of 6 weeks. The class met daily for 41 min and students used this time to work at their own pace, on their own netbooks, to complete their digital storytelling project. Each week, a new task involved in the digital storytelling production process was introduced by either Ms. Lane or the researcher who modelled and explained the task during whole class demonstrations. While the students were independently working on their projects at their own pace, the classroom teacher and researcher were able to provide students with one-on-one technological scaffolding as required during the creation process. Students appeared to enjoy and appreciate the focused attention on their work. There was no expectation that students would work on their projects outside of this class time though many of them did (per student report).

In week 1, Ms. Lane and the researcher introduced the project and showed digital storytelling examples. Most of these examples were drawn from the Storycenter’s website as they fit with the parameters we were using in the classroom. These parameters include a 3–5-min target length and the use of still images. Prior to the project, both Ms. Lane and the researcher created digital stories using the same written instructions and prompts provided to the students. Their digital stories were also shared with the class as examples.

Students were offered the following digital storytelling prompts:

- Tell a story that illustrates how you think your high school education has/has not prepared you for your life beyond high school.
- Tell a story that illustrates why you think high school education is/is not important.
- Tell a story that illustrates your best or worst moment/event at school and the ways that this experience has shaped you.
- Tell a story that illustrates something high school administrators, teachers and policy makers need to know about you in order to make high school a better place for other students
- Tell a story that illustrates the ways school does/does not reflect your life outside of school and why this does/does not matter

Initially, students began brainstorming their ideas and developing their digital storytelling scripts using pen and paper and working in small groups. Students did not have access to their netbooks during the first week while Ms. Lane and the school’s technology support person downloaded Movie Maker software onto their netbooks. Students with Individual Education Programs accessed their additional tutoring support and the researcher also worked with the majority of the students to develop their ideas and draft their initial scripts. The researcher’s feedback generally centred on either encouraging the students to expand their story by adding pertinent details or helping students narrow the focus of their stories, sticking to one main narrative thread.

In week 2, students typed up their stories and continued to work on their story scripts. The researcher continued to provide individual consultations with students about their digital stories as required throughout this process. In one particular case, the researcher actually typed the story for the student so they were able to move on to editing phase of the process.

In week 3, Creative Commons^7^ was introduced as a source of licenced images for use in their projects. We discussed issues of copyright and the importance of using pictures to add depth and meaning to their narrative. Students began to search for and select their images. Many students also selected and included photos of their families and communities.

In week 4, Ms. Lane and the researcher demonstrated how to use Movie Maker software to import audio and capture images. The students then began to record their stories onto their netbooks and import their narration and images into their story boards. We also introduced Jamendo,^8^ a site that has free music, which is available for sharing and use as soundtracks. At this time we once again discussed issues of copyright and the problems inherent in using their favourite songs as background music. It should be noted that only a few students took these discussions of copyright issues on board, and this would be an area for emphasis for future projects.

In weeks 5 and 6, students edited and fine-tuned their digital stories and then completed a reflection about the final product. In week 7, Ms. Lane moved onto another project and there was no additional class time available for this work. To receive course credit, students had to submit their digital stories and their reflections by the end of the term. All the stories were shared with school administration, but not with their peers (by student request).

For the most part, these digital stories seemed intended for an unfamiliar audience, although several were directed at the school staff and administration. This may have been because of the prompts, or because the digital stories were a school assignment and students knew they would be viewed by Ms. Lane and the principal. Perhaps if the project included a community or classroom event where the stories were viewed publically this would have shaped the way the stories were framed and the kinds of stories that were told.

Students refused a whole class viewing that was suggested at the onset of the project. Ms. Lane and the researcher agreed, largely because we were taken aback with the student responses to the initial class assignment which asked students to write about a time they had overcome adversity. This work and the stories that followed that semester revealed some expected and many unexpected social issues including bullying, parental abandonment, parental drug use, abuse and a boyfriend’s death by autoerotic asphyxiation. Many of the students revealed a relatively low social status in the school environment.

### Which stories?

This research is a part of a much larger study which was designed as a narrative inquiry (Clandinin & Connelly, 2000), and which the digital stories were one component. At the heart of narrative inquiry is the belief that story is a human instinct; stories are a form of communication and they reveal beliefs, values and culture. They also serve as temporal and geographical markers. Thus, narrative is a powerful research tool. Stories can come from events that have already taken place, but often times they also guide behaviour and shape the events that will come (Frank, 2010). For many of these students, they were living stories and narratives being told about them as “non-university-bound youth”. Narrative inquiry is an important research method, because as listeners and researchers we come to care about the story and by extension the storytellers (Bochner, 2014), in this case, rural youth. Narrative inquiry was used as a way to make sense of and understand lived experiences (Clandinin & Connelly, 2000) of rural students growing up within their community amidst a plethora of expectations about who they would and would not become in the future.

Twenty-five students consented to participate in this research prior to the onset of the study, one consented at the end, proud of her digital story and hoping it would be shared. Three students left school or did not complete the course and were not included in the data analysis. For the larger project, the digital stories as well as student work, and interviews were examined to identify the themes and patterns that emerged across these texts (See Staley, 2017). Here, only the digital story texts of the 23 students who consented to participate in this research, remained in school for the duration of the project, and completed their digital stories were analysed thematically (Pinnegar & Hamilton, 2011) and we looked explicitly at their talk about the future. This is the data presented here.

## Results

In their digital story texts, these students talk about the future in two main ways. Firstly, they talk about their future, aspirations, dreams and hopes. Secondly, they talked about how school could be better. We have interpreted this to mean, how school could be better *in the future, for future students.* Examples of each of these kinds of future talk are provided in the following paragraphs.

### Using digital storytelling to consider their future

Claire was a bright and approachable 18-year-old female student with a partial scholarship to complete an 18-month cosmetology qualification in a nearby town. Claire planned to attend the program with one of her childhood friends in lieu of university. Upon graduation, Claire intended to join her family’s business as a practicing cosmetologist. Her digital story was one which explicitly revolved around high school and its relationship to her future. Claire’s digital story text is acerbic and begins as such:

University. The ‘ideal’ destination for Shady Grove students after high school. That’s where they want us all to go. University is the answer to all our questions: What should I do after high school? What career do I pursue? How do I pick my career? How do I get a good job? University is always the answer they say.

Discussion of the future was not unique to Claire. Paul’s digital story stated, “when you first enter high school you become pre-programmed like a robot to only worry about one thing: university.” He goes on to say, “the stress destroys most kids’ dreams,” and many students “lose their sense of freedom, they become these mindless robots.” Both Claire and Paul were quite forceful in their choice of words and images in their digital stories as they communicated the pressure they felt around university attendance, and the criticism they received when they articulated a desire for alternative career pathways.

For many of these non-university-bound students, their stories indicate that the school’s focus on university track professions and subsequent dismissal of plans that deviate from university entrance, often undermines student’s (equally worthwhile) dreams and aspirations. For example, Claire’s stated dream is actually to run her own business (like her father); another student Ben was explicit that “I would like to be a professional hunter.” Joe was less clear when he indicated, “I would like to do something with construction but I’d like doing something like a park ranger or a wildlife officer.” Because of the university focus in the high school curriculum, it appeared that the variety of vocations available to a young person were not explored, such that student’s future aspirations and the pathways for those career choices could be pursued.

Susan noted “as a high schooler I have no real idea of what I want to do after school and I feel that sometimes they push university… on you as a student.” As Claire’s quote above suggests, Claire’s digital story, in particular emphasised how she and many of her peers were not encouraged to think about their aspirations and desires, if those were not directly related to university attendance.

Unsurprisingly, many of the students in this research, Claire included, were ambivalent about school and its usefulness and purpose in their life. Chad selected words like “worthless” and “pointless” to talk about his high school education. He wondered, “why would I ever, ever, ever, need to find out the square root of 257? I don’t care what the square root is. I know what the square root of Algebra is, it’s boring.” Ben, was also quite outspoken about the futility of school (e.g. his digital story opened with “I think that school is worthless”) and he tells the listener,

We want to be doing stuff that we are actually going to be doing with our lives. Meeting the needs of students should be a priority. The teachers and administrators should be asking the students what is it you are wanting to do, why they are choosing this and how should we form classes around these preferences?

Sam (who would attend a 4-year college to study journalism the following year despite his participation in this course) had a different take on the school’s approach to university. In his digital story he revealed:

The students in school now are the leaders of tomorrow and need to be ready to make that next step towards university and I think that if students are getting bored in class then they are going to be more likely not to be interested in going to university... students will think that since high school was boring that university will be just like it.

Insights from these digital stories speak to the potential benefits of student perspectives, particularly in regard to knowing and understanding what students want for their lives, so curricular opportunities, encouragement and pushing to excel can align (rather than conflict) with student’s aspirations (Sizer, 1996). We see this also in the stories where students focus was not on their future desires, but the changes they would like to see in their high school.

### Using digital storytelling to consider school improvements

Many digital stories contained comments which fell into a thematic category we have termed “school improvements.” Invariably, students wanted learning that was “hands on”; they wanted educators who “get more involved” and were “not mean.” They also wanted “more responsibilities” which might be interpreted as more meaningful engagement with curricular content. Across the stories, some of these suggestions were quite general and some were quite specific. For example, Jake dislikes school generally because, “you get yelled at for every little thing and you can’t have fun because when you’re having fun in school the teachers get mad and yell at you because they’re not having fun.” Jake dislikes school specifically because “the school is ripping us off” by charging $2.25 for a slice of pizza and a salad.

Karen’s story was unique in that it explicitly put her in the position where she had the power to run the school. She begins her digital story with “I would throw out the handbook, burn it, and then I would sit back in that really nice chair [the principal] sits on daily and just sleep the rest of the day.” Other actions include abandoning the school dress code, and cancelling school whenever she feels like it. Karen follows this up by saying,

My students honestly wouldn’t get that much education. But for the most part they would have a 10^th^ grade education level by the time they graduated. Because since we pretty much learn the same stuff over and over again each year.

Further, Karen has a definite belief that she is powerless within the school system. In her digital story, she talks about the changes she would like to make to graduation requirements, particularly the community service component. Here she compares her relationship to the school with that of prisoners of the state when she says:

I personally think we shouldn’t have to serve our community just to graduate, I mean that’s what they have all the criminals do is community service, and I’m pretty sure that there are enough criminals to go around and do some community service.

It is apparent that the purpose of community service has not been made clear to Karen; it is simply another task to be completed before she is allowed to leave school. Karen concludes that if she was indeed running the school, she would “make sure that the kids that go here would actually look forward to getting up every day…” and “they would like their teachers.”

Students’ digital stories point to the changes that they would like to see in schools, and there are some recurrent themes including curricular “usefulness” (e.g. “I think we should only have classes that are helpful to the careers we want”), teacher relationships (e.g. “teachers need to have a positive attitude towards teaching the students”) and participation (e.g. “let the students be more active”).

In wishing for changes in schooling, students indicate that they care about their education and that they are seeking an education where they get value for their time and effort. At the moment, as with the school pizza, they feel they are getting “ripped off.” From their perspectives, they are being made to get up very early and do a lot of work, for little reward.

These students reported a desire to be successful in their individual careers, and yet as non-university-bound students, they have found themselves in this category that is a low priority for their educators. As a final example, Lachlan, who desires to be a tattoo artist, revealed he was unable to get into a single art class in his time at Shady Grove High School. While his selected career might not be well regarded in a school setting focused on higher education, surely engaging Lachlan in his passions and talents might give him the best opportunity to become an artist whatever the form and function.

## Discussion

The theme of “future” was frequent in student’s digital stories. Students wrote both about their aspirations, and the kinds of things they would like to see educators and administrators doing to make school more meaningful for them. As a way to frame our discussion of the students’ digital stories, we use Smyth’s (2006) learner-centred policy constellation.

**Fig. 1.**
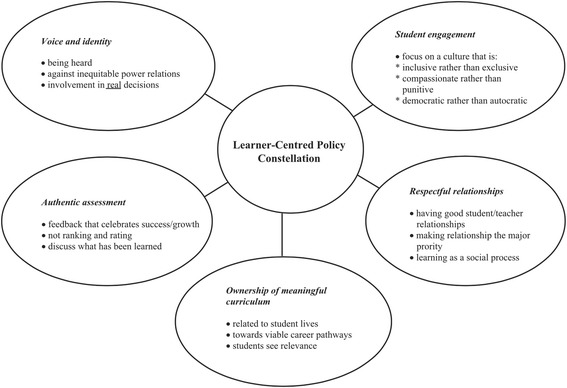
Learner-centred policy constellation

Smyth’s (2006) work is concerned with the growing numbers of disengaged students who are at risk of dropping out, because they have concluded schooling “is not for them” and posits a major shift in policy to better account for all learners, including those currently marginalised by the system. Smyth (2006) presents his learning constellation as a way forward for re-envisioning education that unshackles itself from the accountability-reform movements of the past. See Fig. 1.

We have used Smyth’s (2006) constellation framework and applied it to this project, demonstrating how educators can implement learner-centred pedagogies with digital storytelling. Using this framework, and the students’ stories, we describe how technologies provide differentiated learning opportunities, so that students could pursue personally relevant topics, which in turn created a meaningful curricular opportunity.

### Voice and identity

In the last 20 to 30 years, the construct of “identity” has emerged as a prominent factor that influences patterns of engagement, academic success and failure among marginalised students (Cummins et al., 2015). Accordingly, schools and educators could strive to provide pedagogical opportunities that recognise student’s culture, language and identity (e.g. Kinloch, 2012; Lee, 2007; Morrell, 2008). Despite the substantial literature on the topic, the constructs of identity and engagement have been virtually absent from “mainstream” research literature and policy discussions concerning educational reform and school achievement (Cummins, Hu, Markus, & Montero, 2015).

Cummins et al. (2015) describe how the creation of multimodal narrative texts enabled Aboriginal students in Canada to begin to unravel the colonising processes at play in their lives. While this project was undertaken with white rural youth from the USA, as seen with Karen’s digital story, this project provided the students with an opportunity to reflect on their position within the education system, their futures and the power dynamics at play. In doing so, the findings revealed that students expressed a strong sense of disconnection and disaffection with the current state of their education. In various ways, we seen them railing against inequity and demanding to be involved in decision making about their lives and learning.

This project privileged their voices by providing them with an opportunity to be heard by the teacher and ultimately by the administration. The high completion rate of digital stories by students previously identified as “disengaged” indicates that they valued the opportunity to express their reflections through the creative student-centred medium of digital storytelling.

### Ownership of meaningful curriculum

A global educational focus on standards and assessment, literacy and numeracy, has led to a narrowing of school curriculums and has seen an increase in the proportion of teenagers classified as “disengaged”. Cothran and Ennis (2000) report that “the number of disengaged students may exceed two thirds of the high school population” (p. 106). Smyth (2006) states,

the reasons students withdraw from school emotionally, educationally, psychologically, and, eventually, physically are multi-faceted and complex, but in the end they boil down to ‘political’ reasons - that is to say, students refuse to make the emotional and relational investment necessary to become engaged with the social institution of schooling in a manner necessary for the learning to occur (p. 288–289).

Erickson’s work (1987) also suggests that these students are not just disengaged but actually developing and pursuing an oppositional identity, at odds with the identities being proffered by the school. This stance is an active refusal to accept the negative identity assigned by the school, and is formed in protest, due to a disconnection between academic material and student’s lives. That is, students protest by refusing to learn (Erickson, 1987).

Cummins (1986, 2001, 2015) argues that marginalised students will succeed educationally to the extent that the patterns of interaction in school reverse those that prevail in the (dominant) society at large. It therefore follows that unless students are presented with an education that’s relevant and affirming of their current and future identities, these students will refuse to invest the energy and commitment necessary for learning to occur.

If students’ educational success is dependent on the school creating an environment which mitigates the marginalising forces of the dominant society, then creating an empowering education for students is dependent “upon the extent to which educators, both collectively and individually, redefine their roles with respect to minority students and communities” (Cummins, 1986, p. 19).

Educators might reflect on how their students’ identities are negotiated in the classroom and community, and attempt to provide learning experiences that simultaneously affirm students’ beliefs and values, while challenging uncontested stereotypes and narrow world views. Without active discussion centring student identity and local issues of importance, wide segments of the high school student community may continue to disengage, choosing to pursue an oppositional identity, rather than one assigned by teachers and school administrators.

Even though the students described here are white, and in the racial majority of their community, they are disenfranchised as students by the school’s firm and fixed focused on university preparation. The students themselves reveal that high school is viewed as a stepping stone or bridge to higher education (a place they are not going) and thus renders their education “worthless” and irrelevant (Staley, 2017).

Education research has demonstrated that young people engage in their schooling in productive and meaningful ways when they can see the connection between their learning and their lives (Delpit, 2002; Lee, 2007; Mitchie, 2009; Kinloch, 2010). If we want students to view high school as a meaningful, relevant educational site that can positively capitalise on their interests, educators might reflect on how meaning is assigned to activities by re-evaluating the goals and purposes of education for non-university-bound students like Claire and her peers.

There is virtually universal agreement among reading and learning theorists that effective instruction involves activating student’s prior knowledge, and it is widely accepted that a learner’s knowledge of the world provides a basis for understanding, learning and remembering facts and ideas (Echevarria, Vogt, & Short., 2016). Therefore, the more educators can contextualise their teaching programs to create locally distinctive learning for marginalised students, the higher their language and literacy outcomes are likely to be, and the more likely they are to see their schooling as relatable and relevant.

For the students in this study, the relevance of high school and the materials being taught there were popular topics for discussion. Overall, the students agreed that school could be more useful to them, as it was hard to see how high school was applicable to their future aspirations. Hardre, Sullivan, & Crowson., (2009) reported that rural students who could see the “usefulness and value” of school learning, had a greater tendency to “exhibit an interest in school, put forth effort, and exhibit intentions to graduate and go on to postsecondary opportunities” (p. 13). That is, if students perceive school and curricular content as “useful” towards their future career pathways, then they are also much more likely to do the very thing school personnel want from them (e.g. study). The significance of these findings is that perceptions of “usefulness” are malleable in comparison to other facets of the education system (see also Irvin, Meece, Byun, Farmer, & Hutchins., 2011).

The digital storytelling project was not seen as useful per se, but students did appreciate the opportunity to share their opinions and stories and choose the topic for exploration. There were some surprisingly personal stories that talked about meaningful experiences, tight family bonds and hope for the future.

### Respectful relationships

Woven into students’ stories was a desire for supportive teacher relationships. Students make clear that they want to like their educators and they want to be liked by them. For example, Sam thought, “the teachers need to have a positive attitude towards teaching the students and not be mean and make students not want to be at school.” Claire felt deeply conflicted when her career plans were not supported by educators and the school guidance counsellors. Ben wanted teachers to ask students “what is it you are wanting to do?” and act on the answers that students provided. Other students simply wanted their educators to be nice, interested and involved in their lives. The digital storytelling project gave Ms. Lane a way into the students’ lives and enabled her to learn much more about her students than she had previously. In many ways, these digital stories became conversation starters for larger discussions about education and life beyond high school. Students were choosing to reveal themselves in various ways.

### Student engagement

The interpersonal power relationships between marginalised students and their educators often reinforce the broader societal patterns of exclusion and discrimination (Cummins, 2001). Smyth’s (2006) framework asserts that effective student engagement develops in classrooms with inclusive, compassionate and democratic cultures (and educators). The implementation of the digital storytelling approach aligned with Smyth’s (2006) prescriptions for engagement because it privileged students’ voices and perspectives. The students responded by engaging in critical conversations and completing set tasks on time, behaviours that were reportedly uncharacteristic for this student cohort. These observations are consistent with the findings of Sleeter’s (2011) review of the outcomes of culturally responsive education programs in the USA. Sleeter’s (2011) synthesis reported that literacy approaches that challenge the operation of coercive relations of power in the wider society produce positive outcomes for students from socially marginalised communities.

Engagement not only improves students’ perceptions of schooling and themselves as a learner, but also there is persuasive international evidence that literacy attainment is enhanced when students engage actively in literacy activities, both in school and in out-of-school contexts. Although much of this research has focused on reading, Cummins (2015) broadens the focus from reading engagement to literacy engagement, which seems appropriate given the considerable research documenting how developing writing expertise plays in improving reading comprehension (Graham & Herbert, 2010).

Here we seek to broaden the focus of engagement further from *literacy* to *language and digital literacies* through the creation of digital stories. This broader stance recognises there is now sufficient evidence to support the inclusion of oral language as a genuine contributor to reading comprehension, the ultimate goal of reading (Konza, 2014), and further, that much of the text that twenty-first century students communicate in and through involves digital modes of reading and writing (or literacies).

### Assessment of digital literacies

The digital stories themselves and the reflections the students produced about the digital storytelling process provided Ms. Lane the opportunity for assessment of the students’ abilities to engage with digital literacies. Students generally reported enjoying the project and displayed some pride in their finished product.

The process of creating digital stories provided students with multiple opportunities to succeed. They first wrote their texts, which were read and collaboratively reviewed and often co-edited for grammar, spelling and content as needed. No tension was noted around editorial comment or suggested changes.^9^

There were multiple chances for students to read their texts for recording onto their digital storyboard. The researcher sat with many of the students as they recorded their stories, and there were certainly students who struggled to decode their own written texts. However, because there was no live audience, students had the chance to practice until they were satisfied with their recorded narrative. Utilising multi-modal literacy skills was a natural by-product of this task, because of the nature of the assignment. What is more, there is procedural knowledge and terminology, including discipline-specific vocabulary that students had to engage with in order to complete their projects.

In the production of their digital stories, students had varied opportunities for edits, and the teacher had many opportunities for input, including extending the student’s thinking and improving literacy practices. Among the tasks completed, students had to storyboard a text against images, often overlaid with selected soundtracks in addition to their narration. This was a complex time-bound technological task which would be valued in many contemporary workspaces.^10^ This is exactly the type of meaningful assignment students reported they would like to see in their classes and gave them a chance to practice the kinds of skills that might be an asset in the future.

### Implications for educators

The students in this project did not see high school as relevant. They did not see value in it, it did not affirm their identity and it was not extending their learning. As we have illustrated using Smyth’s (2006) constellation, a digital storytelling project can be an effective approach to engaging students, providing a fertile space to develop teacher-student relationships and invite student input into the design of relevant learning materials and their future education pathways.

The intent of this paper is to encourage educators to expand their pedagogies to include digital storytelling. Digital storytelling demands students use literacy skills inherent in the curriculum, and it taps into the rich potential of story, which provides students a forum for expressing themes and topics close to their hearts. This in turn gives educators an opportunity to get to know their students (and students indicated that they want to be known). The creation, production and sharing of stories can provide a way to link the curriculum, what students think they *do* in school, with their visions for their future. Digital storytelling thus has the opportunity to be a “living text” (Morrell, 2008, p. 170) and provide what Harwood et al. (2015) call “windows of aspiration.”

There are two salient issues we have identified with this project. The first is that this work would have much better served the students, teacher and school administration if it happened in the first year of high school rather than the last. It was a bit late to identify and meet the needs of students when they only had 4 months of high school remaining.

The second issue is related to technological access and support. To complete this project in the 6-week time frame, there were two relatively tech savvy adults working regularly with a class of 25 students, and both the teacher and researcher were adept at utilising the technologies necessary to create a digital storytelling project. Further, each of the students had school-issued laptops with internet access, so they were able to work independently on their projects both during class time and at home (if necessary). Digital storytelling projects are time intensive and would certainly be harder to manage using shared computing facilities.

## Conclusions

This research points to the necessity for educators to ask students to share their stories. The stories created by Shady Grove’s high school students lead us to conclude that the production and sharing of stories can encourage students to reveal and complicate narratives they have of themselves, and others have of them. Further, that digital storytelling provides students an opportunity to not only to tell their stories, but also to nuance their perspectives. In this way, students might be encouraged to engage in critical self-reflexive work as they interrogate power relations within the context of the curricula and the school.

Throughout the analysis of data, there were notable and insistent demands that educators do a better job of getting to know and understand students and their aspirations. We need educators and administrators to know their students and account for their future aspirations so that the high school years can be a time for students to hone their skills and passions in ways that will be useful to their future selves. Through digital storytelling, and through the exploration of students’ futures, we might collectively re-imagine schools as places that support, inspire, and nurture what students want and need, by including all stakeholders—including students—in educational conversations (Fine & Weis, 2003).

There was an opportunity in this research for students to question the role of education in their lives so that schooling serves not as “wasted time” but as an opportunity for students to see what is possible. Further, the digital production of these stories gave students an opportunity to share their ideas globally as “netizens”^11^ (Parker, 2014), if they chose, providing them with the potential to connect with likeminded peers beyond the rolling green hills of the Midwestern USA.

So we conclude with the words of Claire, “I refuse to fall onto the ideal path of so many others when I know it’s not right for me… Why does it seem like we are being pushed and limited…?” Perhaps it is time to re-envision what high school might be for non-university-bound high school youth. By harnessing technology and involving students in work which includes critical examination of the educational supports and limitations students perceive in their lives, perhaps high schools can better serve this population so that future students when asked will say: high school is useful, engaging, and pushes me to become who I want be.

## Footnotes

1 Rather than the digital storytelling movement

2
www.storycenter.org

3 The rural-fringe designation means that the school is located in a “territory that is less than or equal to 5 miles from an urbanised area, or a rural territory that is less than or equal to 2.5 miles from an urban cluster” (Definition from http://nces.ed.gov/surveys/ruraled/definitions.asp retrieved 1/24/14).

4 All names are pseudonyms

5 Ms. Lane’s perceived success of the project meant she continued to teach *Journey Beyond Shady Grove*, with the inclusion of the digital storytelling project in the following school years. One impact of her experience with this research collaboration was that she left Shady Grove in 2014 to pursue her PhD.

6
https://www.storycenter.org/

7
www.creativecommons.org

8
www.Jamendo.com

9 Perhaps because feedback was individual and provided in small ways throughout the project, students were receptive to editorial suggestions. Perhaps also, they complied with suggestions because they viewed the researcher and teacher as the authorities who would ultimately grade their efforts. Possibly, because they were in 12th grade, with months until graduation, the students did not really care about the class nor the project and participated willingly simply as a way to pass time until they were “free”.

10 In this course, students also created resumes and a profile on a job-seeking website and were able to cite their digital stories as an example of digital capability if they chose to. At least one student applied for work and was employed as a result of this classroom activity.

11 citizens of the Web
